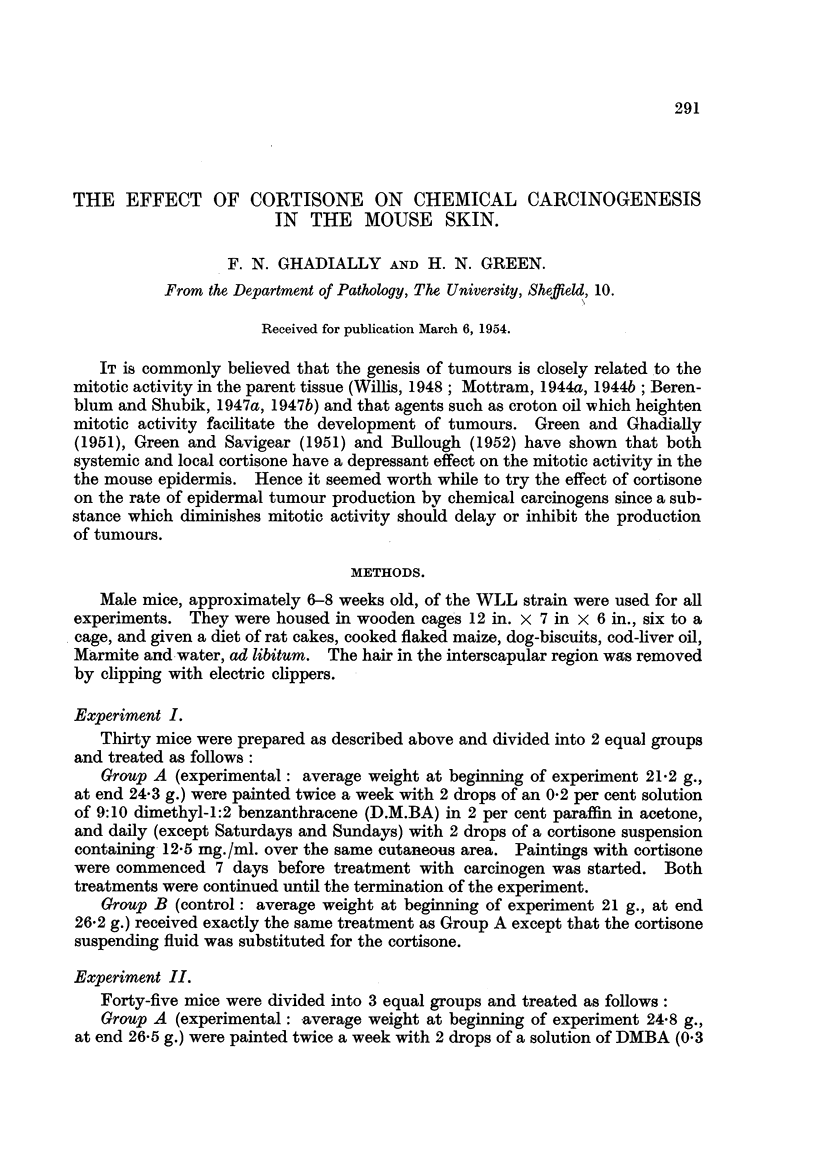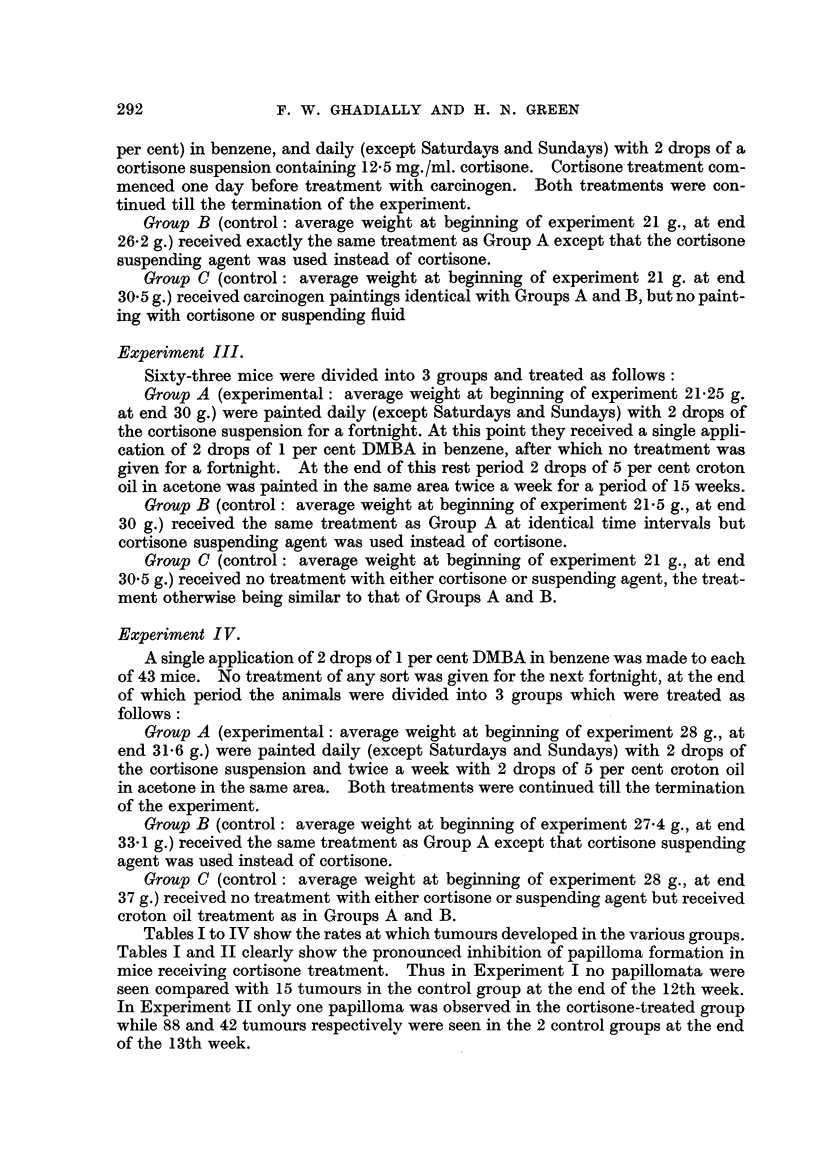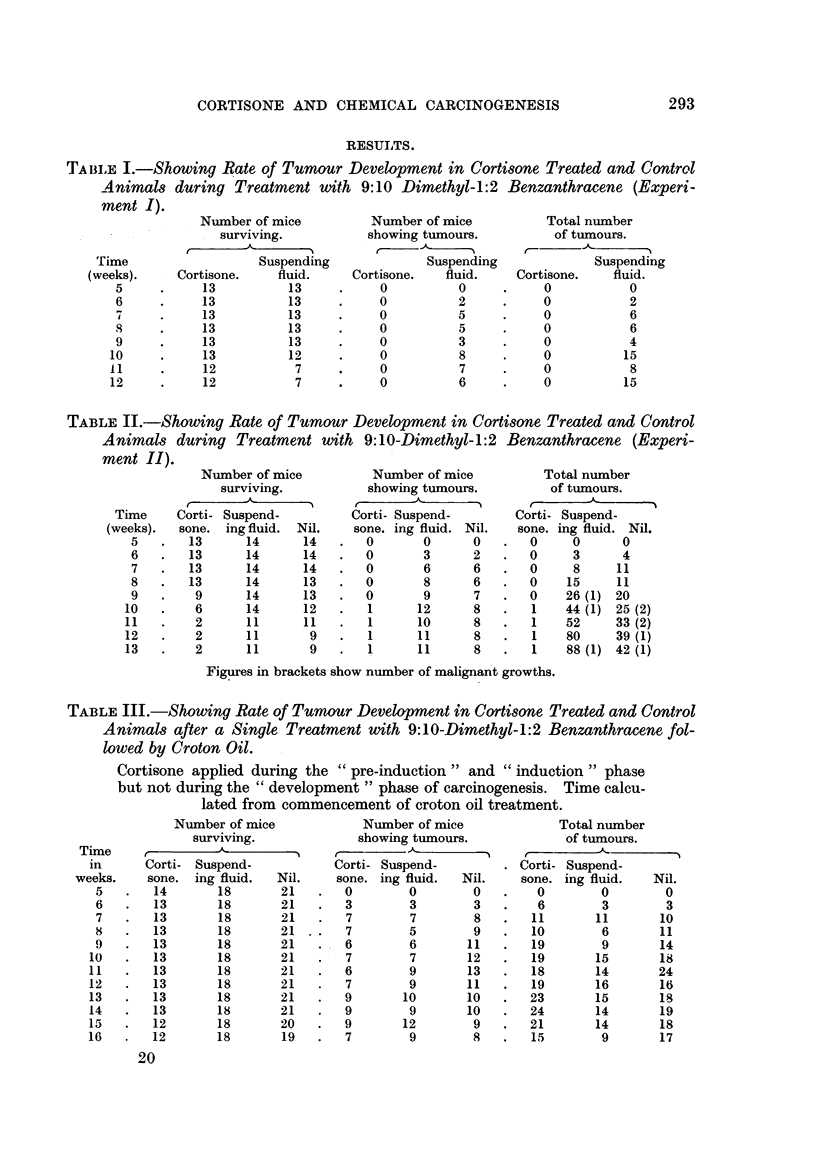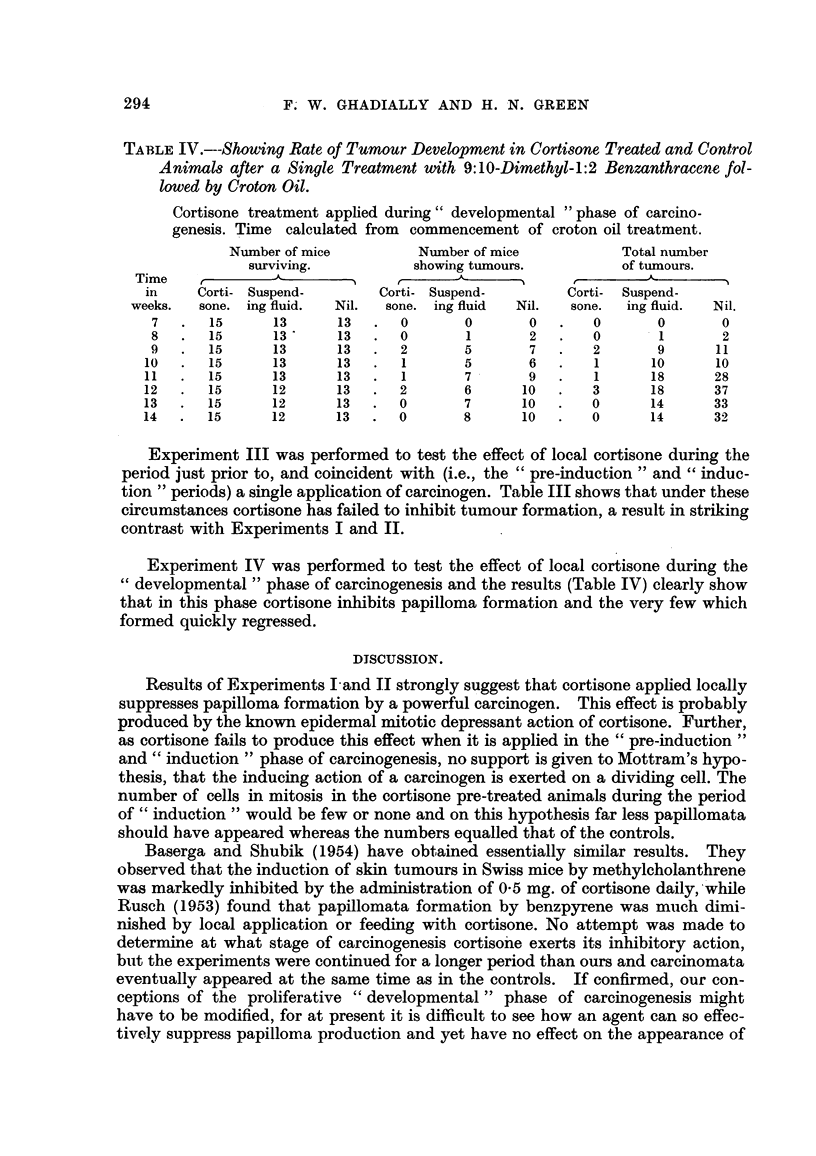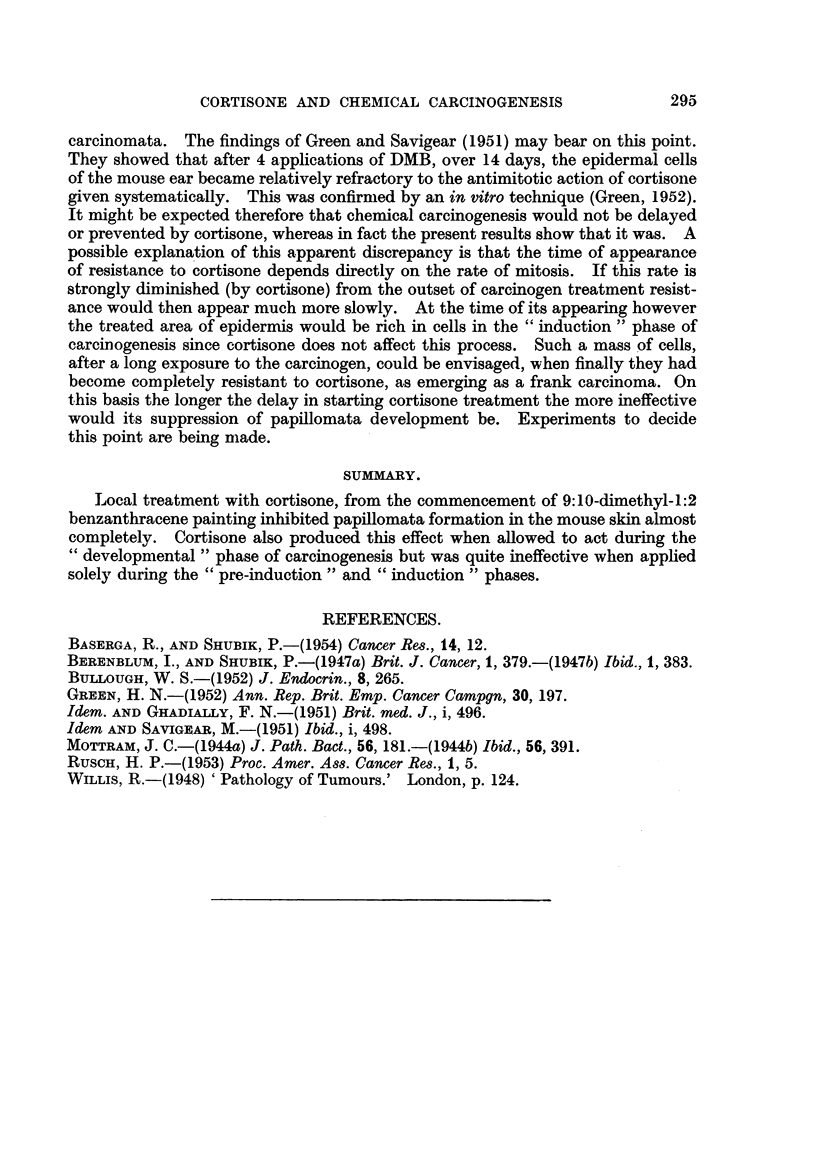# The Effect of Cortisone on Chemical Carcinogenesis in the Mouse Skin

**DOI:** 10.1038/bjc.1954.29

**Published:** 1954-06

**Authors:** F. N. Ghadially, H. N. Green


### THE EFFECT OF CORTISONE ON CHEMICAL CARCINOGENESIS IN THE MOUSE SKIN.

F. N. GHADIALLY AND H. N. GREEN.

*From the Department of Pathology, The University, Sheffield, 10.*

Received for publication March 6, 1954.

IT is commonly believed that the genesis of tumours is closely related to the mitotic activity in the parent tissue (Willis, 1948 ; Mottram, 1944*a*, 1944*b* ; Berenblum and Shubik, 1947*a*, 1947*b*) and that agents such as croton oil which heighten mitotic activity facilitate the development of tumours. Green and Ghadially (1951), Green and Savigear (1951) and Bullough (1952) have shown that both systemic and local cortisone have a depressant effect on the mitotic activity in the the mouse epidermis. Hence it seemed worth while to try the effect of cortisone on the rate of epidermal tumour production by chemical carcinogens since a substance which diminishes mitotic activity should delay or inhibit the production of tumours.

### METHODS.

Male mice, approximately 6–8 weeks old, of the WLL strain were used for all experiments. They were housed in wooden cages 12 in. × 7 in × 6 in., six to a cage, and given a diet of rat cakes, cooked flaked maize, dog-biscuits, cod-liver oil, Marmite and water, *ad libitum*. The hair in the interscapular region was removed by clipping with electric clippers.

### *Experiment I.*

Thirty mice were prepared as described above and divided into 2 equal groups and treated as follows :

*Group A* (experimental : average weight at beginning of experiment 21·2 g., at end 24·3 g.) were painted twice a week with 2 drops of an 0·2 per cent solution of 9:10 dimethyl-1:2 benzanthracene (D.M.BA) in 2 per cent paraffin in acetone, and daily (except Saturdays and Sundays) with 2 drops of a cortisone suspension containing 12·5 mg./ml. over the same cutaneous area. Paintings with cortisone were commenced 7 days before treatment with carcinogen was started. Both treatments were continued until the termination of the experiment.

*Group B* (control : average weight at beginning of experiment 21 g., at end 26·2 g.) received exactly the same treatment as Group A except that the cortisone suspending fluid was substituted for the cortisone.

### *Experiment II.*

Forty-five mice were divided into 3 equal groups and treated as follows :

*Group A* (experimental : average weight at beginning of experiment 24·8 g., at end 26·5 g.) were painted twice a week with 2 drops of a solution of DMBA (0·3

per cent) in benzene, and daily (except Saturdays and Sundays) with 2 drops of a cortisone suspension containing 12·5 mg./ml. cortisone. Cortisone treatment commenced one day before treatment with carcinogen. Both treatments were continued till the termination of the experiment.

*Group B* (control: average weight at beginning of experiment 21 g., at end 26·2 g.) received exactly the same treatment as Group A except that the cortisone suspending agent was used instead of cortisone.

*Group C* (control: average weight at beginning of experiment 21 g. at end 30·5 g.) received carcinogen paintings identical with Groups A and B, but no painting with cortisone or suspending fluid

*Experiment III.*

Sixty-three mice were divided into 3 groups and treated as follows:

*Group A* (experimental: average weight at beginning of experiment 21·25 g. at end 30 g.) were painted daily (except Saturdays and Sundays) with 2 drops of the cortisone suspension for a fortnight. At this point they received a single application of 2 drops of 1 per cent DMBA in benzene, after which no treatment was given for a fortnight. At the end of this rest period 2 drops of 5 per cent croton oil in acetone was painted in the same area twice a week for a period of 15 weeks.

*Group B* (control: average weight at beginning of experiment 21·5 g., at end 30 g.) received the same treatment as Group A at identical time intervals but cortisone suspending agent was used instead of cortisone.

*Group C* (control: average weight at beginning of experiment 21 g., at end 30·5 g.) received no treatment with either cortisone or suspending agent, the treatment otherwise being similar to that of Groups A and B.

*Experiment IV.*

A single application of 2 drops of 1 per cent DMBA in benzene was made to each of 43 mice. No treatment of any sort was given for the next fortnight, at the end of which period the animals were divided into 3 groups which were treated as follows:

*Group A* (experimental: average weight at beginning of experiment 28 g., at end 31·6 g.) were painted daily (except Saturdays and Sundays) with 2 drops of the cortisone suspension and twice a week with 2 drops of 5 per cent croton oil in acetone in the same area. Both treatments were continued till the termination of the experiment.

*Group B* (control: average weight at beginning of experiment 27·4 g., at end 33·1 g.) received the same treatment as Group A except that cortisone suspending agent was used instead of cortisone.

*Group C* (control: average weight at beginning of experiment 28 g., at end 37 g.) received no treatment with either cortisone or suspending agent but received croton oil treatment as in Groups A and B.

Tables I to IV show the rates at which tumours developed in the various groups. Tables I and II clearly show the pronounced inhibition of papilloma formation in mice receiving cortisone treatment. Thus in Experiment I no papillomata were seen compared with 15 tumours in the control group at the end of the 12th week. In Experiment II only one papilloma was observed in the cortisone-treated group while 88 and 42 tumours respectively were seen in the 2 control groups at the end of the 13th week.

### RESULTS.

TABLE I.—*Showing Rate of Tumour Development in Cortisone Treated and Control Animals during Treatment with* 9:10 *Dimethyl*-1:2 *Benzanthracene* (*Experiment I*).

| Time (weeks). | Number of mice surviving. Cortisone. | Suspending fluid. | Number of mice showing tumours. Cortisone. | Suspending fluid. | Total number of tumours. Cortisone. | Suspending fluid. |
|---|---|---|---|---|---|---|
| 5 | 13 | 13 | 0 | 0 | 0 | 0 |
| 6 | 13 | 13 | 0 | 2 | 0 | 2 |
| 7 | 13 | 13 | 0 | 5 | 0 | 6 |
| 8 | 13 | 13 | 0 | 5 | 0 | 6 |
| 9 | 13 | 13 | 0 | 3 | 0 | 4 |
| 10 | 13 | 12 | 0 | 8 | 0 | 15 |
| 11 | 12 | 7 | 0 | 7 | 0 | 8 |
| 12 | 12 | 7 | 0 | 6 | 0 | 15 |

TABLE II.—*Showing Rate of Tumour Development in Cortisone Treated and Control Animals during Treatment with* 9:10-*Dimethyl*-1:2 *Benzanthracene* (*Experiment II*).

| Time (weeks). | Number of mice surviving. Cortisone. | Suspending fluid. | Nil. | Number of mice showing tumours. Cortisone. | Suspending fluid. | Nil. | Total number of tumours. Cortisone. | Suspending fluid. | Nil. |
|---|---|---|---|---|---|---|---|---|---|
| 5 | 13 | 14 | 14 | 0 | 0 | 0 | 0 | 0 | 0 |
| 6 | 13 | 14 | 14 | 0 | 3 | 2 | 0 | 3 | 4 |
| 7 | 13 | 14 | 14 | 0 | 6 | 6 | 0 | 8 | 11 |
| 8 | 13 | 14 | 13 | 0 | 8 | 6 | 0 | 15 | 11 |
| 9 | 9 | 14 | 13 | 0 | 9 | 7 | 0 | 26 (1) | 20 |
| 10 | 6 | 14 | 12 | 1 | 12 | 8 | 1 | 44 (1) | 25 (2) |
| 11 | 2 | 11 | 11 | 1 | 10 | 8 | 1 | 52 | 33 (2) |
| 12 | 2 | 11 | 9 | 1 | 11 | 8 | 1 | 80 | 39 (1) |
| 13 | 2 | 11 | 9 | 1 | 11 | 8 | 1 | 88 (1) | 42 (1) |

Figures in brackets show number of malignant growths.

TABLE III.—*Showing Rate of Tumour Development in Cortisone Treated and Control Animals after a Single Treatment with* 9:10-*Dimethyl*-1:2 *Benzanthracene followed by Croton Oil.*

Cortisone applied during the " pre-induction " and " induction " phase but not during the " development " phase of carcinogenesis. Time calculated from commencement of croton oil treatment.

| Time in weeks. | Number of mice surviving. Cortisone. | Suspending fluid. | Nil. | Number of mice showing tumours. Cortisone. | Suspending fluid. | Nil. | Total number of tumours. Cortisone. | Suspending fluid. | Nil. |
|---|---|---|---|---|---|---|---|---|---|
| 5 | 14 | 18 | 21 | 0 | 0 | 0 | 0 | 0 | 0 |
| 6 | 13 | 18 | 21 | 3 | 3 | 3 | 6 | 3 | 3 |
| 7 | 13 | 18 | 21 | 7 | 7 | 8 | 11 | 11 | 10 |
| 8 | 13 | 18 | 21 | 7 | 5 | 9 | 10 | 6 | 11 |
| 9 | 13 | 18 | 21 | 6 | 6 | 11 | 19 | 9 | 14 |
| 10 | 13 | 18 | 21 | 7 | 7 | 12 | 19 | 15 | 18 |
| 11 | 13 | 18 | 21 | 6 | 9 | 13 | 18 | 14 | 24 |
| 12 | 13 | 18 | 21 | 7 | 9 | 11 | 19 | 16 | 16 |
| 13 | 13 | 18 | 21 | 9 | 10 | 10 | 23 | 15 | 18 |
| 14 | 13 | 18 | 21 | 9 | 9 | 10 | 24 | 14 | 19 |
| 15 | 12 | 18 | 20 | 9 | 12 | 9 | 21 | 14 | 18 |
| 16 | 12 | 18 | 19 | 7 | 9 | 8 | 15 | 9 | 17 |

TABLE IV.—*Showing Rate of Tumour Development in Cortisone Treated and Control Animals after a Single Treatment with* 9:10-*Dimethyl*-1:2 *Benzanthracene followed by Croton Oil.*

Cortisone treatment applied during " developmental " phase of carcinogenesis. Time calculated from commencement of croton oil treatment.

| Time in weeks. | Number of mice surviving. | | | Number of mice showing tumours. | | | Total number of tumours. | | |
|---|---|---|---|---|---|---|---|---|---|
| | Corti-sone. | Suspend-ing fluid. | Nil. | Corti-sone. | Suspend-ing fluid | Nil. | Corti-sone. | Suspend-ing fluid. | Nil. |
| 7 | 15 | 13 | 13 | 0 | 0 | 0 | 0 | 0 | 0 |
| 8 | 15 | 13 | 13 | 0 | 1 | 2 | 0 | 1 | 2 |
| 9 | 15 | 13 | 13 | 2 | 5 | 7 | 2 | 9 | 11 |
| 10 | 15 | 13 | 13 | 1 | 5 | 6 | 1 | 10 | 10 |
| 11 | 15 | 13 | 13 | 1 | 7 | 9 | 1 | 18 | 28 |
| 12 | 15 | 12 | 13 | 2 | 6 | 10 | 3 | 18 | 37 |
| 13 | 15 | 12 | 13 | 0 | 7 | 10 | 0 | 14 | 33 |
| 14 | 15 | 12 | 13 | 0 | 8 | 10 | 0 | 14 | 32 |

Experiment III was performed to test the effect of local cortisone during the period just prior to, and coincident with (i.e., the " pre-induction " and " induction " periods) a single application of carcinogen. Table III shows that under these circumstances cortisone has failed to inhibit tumour formation, a result in striking contrast with Experiments I and II.

Experiment IV was performed to test the effect of local cortisone during the " developmental " phase of carcinogenesis and the results (Table IV) clearly show that in this phase cortisone inhibits papilloma formation and the very few which formed quickly regressed.

### DISCUSSION.

Results of Experiments I and II strongly suggest that cortisone applied locally suppresses papilloma formation by a powerful carcinogen. This effect is probably produced by the known epidermal mitotic depressant action of cortisone. Further, as cortisone fails to produce this effect when it is applied in the " pre-induction " and " induction " phase of carcinogenesis, no support is given to Mottram's hypothesis, that the inducing action of a carcinogen is exerted on a dividing cell. The number of cells in mitosis in the cortisone pre-treated animals during the period of " induction " would be few or none and on this hypothesis far less papillomata should have appeared whereas the numbers equalled that of the controls.

Baserga and Shubik (1954) have obtained essentially similar results. They observed that the induction of skin tumours in Swiss mice by methylcholanthrene was markedly inhibited by the administration of 0·5 mg. of cortisone daily, while Rusch (1953) found that papillomata formation by benzpyrene was much diminished by local application or feeding with cortisone. No attempt was made to determine at what stage of carcinogenesis cortisone exerts its inhibitory action, but the experiments were continued for a longer period than ours and carcinomata eventually appeared at the same time as in the controls. If confirmed, our conceptions of the proliferative " developmental " phase of carcinogenesis might have to be modified, for at present it is difficult to see how an agent can so effectively suppress papilloma production and yet have no effect on the appearance of carcinomata. The findings of Green and Savigear (1951) may bear on this point. They showed that after 4 applications of DMB, over 14 days, the epidermal cells of the mouse ear became relatively refractory to the antimitotic action of cortisone given systematically. This was confirmed by an *in vitro* technique (Green, 1952). It might be expected therefore that chemical carcinogenesis would not be delayed or prevented by cortisone, whereas in fact the present results show that it was. A possible explanation of this apparent discrepancy is that the time of appearance of resistance to cortisone depends directly on the rate of mitosis. If this rate is strongly diminished (by cortisone) from the outset of carcinogen treatment resistance would then appear much more slowly. At the time of its appearing however the treated area of epidermis would be rich in cells in the " induction " phase of carcinogenesis since cortisone does not affect this process. Such a mass of cells, after a long exposure to the carcinogen, could be envisaged, when finally they had become completely resistant to cortisone, as emerging as a frank carcinoma. On this basis the longer the delay in starting cortisone treatment the more ineffective would its suppression of papillomata development be. Experiments to decide this point are being made.

### SUMMARY.

Local treatment with cortisone, from the commencement of 9:10-dimethyl-1:2 benzanthracene painting inhibited papillomata formation in the mouse skin almost completely. Cortisone also produced this effect when allowed to act during the " developmental " phase of carcinogenesis but was quite ineffective when applied solely during the " pre-induction " and " induction " phases.

### REFERENCES.

BASERGA, R., AND SHUBIK, P.—(1954) *Cancer Res.*, **14**, 12.
BERENBLUM, I., AND SHUBIK, P.—(1947*a*) *Brit. J. Cancer*, **1**, 379.—(1947*b*) *Ibid.*, **1**, 383.
BULLOUGH, W. S.—(1952) *J. Endocrin.*, **8**, 265.
GREEN, H. N.—(1952) *Ann. Rep. Brit. Emp. Cancer Campgn*, **30**, 197.
*Idem.* AND GHADIALLY, F. N.—(1951) *Brit. med. J.*, i, 496.
*Idem* AND SAVIGEAR, M.—(1951) *Ibid.*, i, 498.
MOTTRAM, J. C.—(1944*a*) *J. Path. Bact.*, **56**, 181.—(1944*b*) *Ibid.*, **56**, 391.
RUSCH, H. P.—(1953) *Proc. Amer. Ass. Cancer Res.*, **1**, 5.
WILLIS, R.—(1948) ' Pathology of Tumours.' London, p. 124.